# The ramification of Cameroon’s Anglophone crisis: conceptual analysis of a looming “Complex Disaster Emergency”

**DOI:** 10.1186/s41018-022-00114-1

**Published:** 2022-01-24

**Authors:** Henry Ngenyam Bang, Roland Azibo Balgah

**Affiliations:** 1 Disaster Management Centre, Bournemouth University, Dorset, Talbot Campus, Fern Barrow, Poole, BH12 5BB UK; 2 College of Technology, The University of Bamenda, P. O. Box 39, Bambili, North West Region Cameroon

**Keywords:** Complex emergency, Anglophone crisis, Cameroon, Human-induced disaster, Complex Disaster Emergency, Human rights violation, Population displacement

## Abstract

One of Africa’s newest struggles for liberation: Cameroon’s Anglophone crisis, which emerged from legal and education grievances in 2016, rapidly escalated into a secessionist political conflict that is threatening the unity of the country, with potential to degenerate into a complex emergency. In an exploratory, qualitative, analytical, and descriptive case study research tradition involving document/content analysis, we apply the Robert Strauss Centre’s complex emergency framework to investigate the potential of the Anglophone crisis, whose ramifications lead us to consider it an acute complex emergency. Our contention is based on the fact that 72.5% of the variables in all the complex emergencies fall within the relevant to extremely relevant ranking criteria. Furthermore, the establishment of a nexus between the Anglophone crisis and a natural hazard-induced disaster suggest an escalation of the crisis to an unbearable level. Using the high probability of a novel eruption at Mt. Cameroon coupled with the eminent threat of the spread of the COVID-19 virus, we contend that unless otherwise, the crisis has immense potential to cumulatively evolve into a “Complex Disaster Emergency” (CDE) in the Anglophone regions of Cameroon. Amidst the existential challenges in providing humanitarian assistance in the conflict region, and by applying the Robert Strauss Centre’s complex emergency framework, this article concludes with an early warning for an impending CDE that could heighten humanitarian challenges unless there is foresight and goodwill by relevant actors to immediately commence a process of adequate contingency planning.

## Introduction

Arguably, human-induced disasters are increasingly at the origin of complex emergencies, given their capacities to rapidly disrupt livelihoods, their multi-causal nature, and the propensity to interrelate political, economic, social, and environmental factors including internal armed conflict (Anderson and Gerber 2018; Culver et al. 2017). Over the past decade, the increasing magnitude of complex emergencies has resulted into dire socio-economic and political consequences posing incredible challenges to humanitarian and emergency response stakeholders (Macias 2013).

Complex emergencies have caused the global population of the forcefully displaced to grow substantially from 43.3 million in 2009 to an unprecedented 70.8 million in 2018 and increasing by 19.2% in 2019 (UNHCR 2019). Developing countries are the most disproportionately affected. Sixty-seven per cent of complex emergencies worldwide occur in Africa where there has been a surge in complex emergencies in the past three decades (Culver et al. 2017). Yet, the capacity and ability of many developing countries to respond to these crises and emergencies have not evolved at an equal pace (Fraser et al. 2017).

Effective emergency response has necessitated collaboration and cooperation, albeit with challenges, between national governments, international organisations, non-governmental organisations, and the civil society to address the problems created by humanitarian crisis and complex emergencies (Streamlau 1998). Often, foreign humanitarian actors are involved only in complex emergencies.

Research on when instabilities (civil unrest, internal crises/conflicts, or emergencies) become complex emergencies is very sparse. Frameworks for disaster management and humanitarian systems have been lopsided in their focus on responding to particular types of crisis/emergencies (Buchanan-Smith and Christoplos 2004) without much thought on the nexus between complex emergencies and major rapid-onset natural hazards/disasters. This article seeks to address these issues by analysing the Cameroon Anglophone crisis.

The Anglophone crisis is one of Cameroon’s several humanitarian crises that has been raging in the country’s North West Region and South West Region—also called the Anglophone region, since 2016. Managing this and other crises has been very challenging to Cameroon’s disaster management frameworks (Bang et al. 2019b). Although Cameroon’s respond to the Anglophone crisis has been facilitated by external humanitarian actors, the response has been largely suboptimal (Craig 2020). A major episode of natural hazard would further complicate the response with potentially awful consequences on the populations in the region.

The article aims to provide an early warning signal of a contemporary emergency of higher magnitude in the restive region if a rapid-onset hazard intersects the Anglophone crisis. The objectives are fourfold: (1) to establish the consequences of the Anglophone crisis, (2) to ascertain the status of the Anglophone crisis as a potential complex emergency, (3) to analyse the risk of an imminent Mt. Cameroon eruption in the crises region, and (4) based on the findings from (1)–(3), to conceptualise a new complex emergency paradigm.

This research can potentially contribute to contemporary international frameworks that provide a roadmap for safer and more resilient communities to crises and disasters. For instance, the 2030 Agenda for Sustainable Development focuses on specific challenges that countries face in pursuit of sustainable development, especially “African countries, least developed countries...as do countries in situations of conflict...” (UN 2015: 7). Furthermore, priority 1 of the Sendai Framework for Disaster Risk Reduction 2015–2030 (UNDRR 2015) emphasises that nations should “develop, periodically update and disseminate, as appropriate, location-based disaster risk information...to decision makers, the general public and communities at risk of exposure to disaster...” (: 15) and (priority 4) to “...further strengthen disaster preparedness for response, take action in anticipation of events, integrate disaster risk reduction in response preparedness and ensure that capacities are in place for effective response and recovery at all levels.” (: 21). This article also seeks to raise consciousness about the challenges involved in operating at the interface between a complex emergency and potential natural hazard-induced disaster.

## Research context and background

### Genesis of the Anglophone crisis

The Anglophone crisis is rooted in Cameroon’s troubled colonial history that eventually gave birth to its dual bi-lingual heritage (French and English official languages). Cameroon became a German colony on July 14, 1884, but after the First World War, during the Versailles treaty in 1919, German Kamerun was forfeited to be administered by Britain and France who carved the territory into two parts: 20% to Britain and 80% to France. The French (Francophone) section achieved independence on January 01, 1960, as *La République du Cameroun* while their English-speaking counterparts (Anglophones) in present-day North West and South West Regions who were under the British administration (Southern Cameroons Trustee Territory) had the option to merge with either *La République du Cameroun* or Nigeria. During a United Nations-organised plebiscite on February 11, 1961, the British-administered Southern Cameroons voted to reunite with the former German Kamerun (French Section) to form the Federal Republic of Cameroon^1^ comprising the states of East Cameroon and West Cameroon (Ngoh 2001).

However, in 1972, Cameroon’s pioneer president (Ahmadu Ahidjo) changed the federal structure of the country in favour of a unitary state called *United Republic of Cameroon*. On February 4, 1984, his predecessor and current President, Paul Biya, further altered the name of the country to *La Républic du Cameroon* (the original name of the French-administered East Cameroon). Since the union of the two Cameroons, the Anglophones who currently constitute around 14% of the population (World Bank 2017) have increasingly complained of being marginalised (Konings and Nyamnjoh 1997). This was compounded by the change of name, viewed by many as an attempt to erase the minority Anglophone identity and forcefully assimilate them into the majority French system. Since independence, the Anglophones have staged several protests against perceived unfair economic and institutional discrimination, marginalisation, and inequality in the appointment of public administrators.^2^ This has often caused a periodic outburst of violence, especially when government forces attempt to repress protesters (Chereji and Lohkoko 2012).

The latest phase of the Anglophone crisis started as peaceful street demonstrations in October 2016 by lawyers’ and teachers’ trade unions, who amongst many grievances, were against the obligatory use of the French language in schools and Law courts in the two English-speaking regions. The government responded harshly by imprisoning the protest leaders and its security forces launched a violent crackdown on protesters. The response was the insurgence of armed secessionist groups since January 2018, who have been demanding the independence of the Anglophone regions aka the Ambazonia Republic. Since then, there have been violent armed confrontations between the secessionist groups and the regular military (HRW 2018; GRID 2019; Lazar 2019).

## Humanitarian crises/emergencies in Cameroon

The Anglophone crisis has added to the other humanitarian emergencies in Cameroon. The mayhem and violence caused by the insurgency of the Sunni Islamist group (Boko Haram) in Northern Nigeria led to the influx of thousands of refugees into Northern Cameroon. Boko Haram’s bloody and violent incursion into Northern Cameroon has caused insecurity with outrageous consequences on the livelihoods and economy in that part of the country (Foyou et al. 2018). In 2020, the terrorist group killed refugees and internally displaced persons in Cameroon’s villages hosting around 18,000 internally displaced persons along the northern border with Nigeria (UNHCR 2020a). Moreover, over 623,400 refugees fleeing intermittent violence perpetrated by armed groups in the Central African Republic since 2013 have settled in neighbouring countries including Cameroon (UNHCR 2020b). These refugees and internally displaced persons have increased the population density in Northern Cameroon, posing a threat to food security, social cohesion, and increased land degradation/erosion in the region (Bang et al. 2019b). These humanitarian crises bear heavily on Cameroon’s capacity to deal with the Anglophone crisis.

## Natural hazard risks in the Anglophone region

**Fig. 1:**
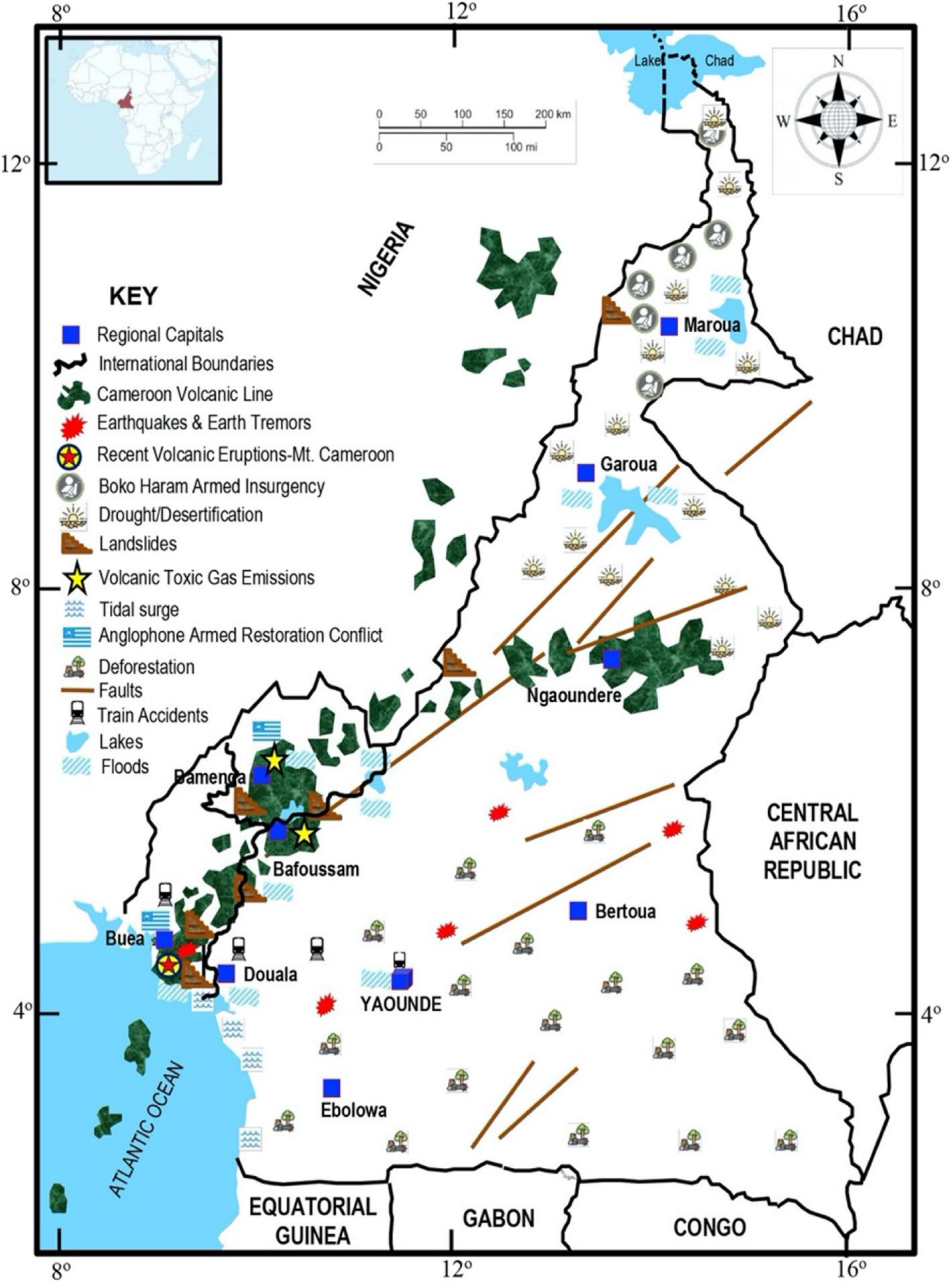
Map of Cameroon showing the Anglophone regions including the main natural hazards in the country. Source: Bang (2021)

Cameroon’s Anglophone region is prone to numerous natural hazards (volcanic eruption, landslides, floods, earth tremors, storms, lightning, toxic gas emission from crater lakes). These hazards are confined to a major topographical feature of geological origin in the country known as the Cameroon Volcanic Line^3^ (Marzoli et al. 2000; Fig. 1). The Lake Nyos Disaster that occurred on August 21, 1986, in Menchum Division (currently a hotspot of the Anglophone crisis) in the North West Region is the worst natural hazard-induced disaster in Cameroon in terms of fatalities. Forcefully released poisonous gasses from Lake Nyos killed 1746 people and 8500 livestock, caused 4500 internally displaced persons, and affected more than 20,000 people (Bang 2012).

Fako Division (another hotspot of the Anglophone crisis) in the South West Region is host to Mt. Cameroon, the most active and largest volcano on the Cameroon Volcanic Line and West/Central Africa. Mt. Cameroon is a huge elliptical stratovolcano with a height of 4095 m above sea level and an area of about 3000 km^2^ that straddles the continental margin at the Gulf of Guinea in the South West Region. The volcanic edifice has a volume of 1200 km^3^, with over 100 craters and cones on its summit and flanks, and is thought to have a near-continuous eruption sequence over the last 10 million years, being most active within the last 3 million years (Marzoli et al. 2000).

Mt. Cameroon is a threat to population centres in the South West Region, where the Anglophone crisis is raging. Past eruptions have caused extensive/expensive damage to property, displaced hundreds of people, and affected thousands more with huge implications for agrarian livelihoods (Favalli et al. 2012; Tsafack et al. 2009). Therefore, knowing when the next eruption will occur is desirable since it may further complicate the existential Anglophone crisis.^4^

## Nexus between political instability and natural hazards

Political instability and/or conflicts negatively impact preparedness, response, and recovery from natural hazard-induced disasters. Several examples abound. An analysis of the interface between political instabilities and natural hazards/disasters in Afghanistan, South Sudan, Colombia, and India revealed how conflicts/political instability aggravated the impact and reduced the coping measures and local resilience capacities to the natural hazards/disasters (Buchanan-Smith and Christoplos 2004). The long-standing conflict in Somalia rendered the government very weak and unable to respond to recurrent flooding and drought (Ferris 2010). Civil unrest in Nepal triggered by squabbles between political parties directly impacted the country’s disaster risk management and response preparedness (Dominguez 2015). Natural hazards can exacerbate humanitarian crises through further displacement of internally displaced persons, massive population displacement, increased destitution for victims, and more pressure on communities hosting internally displaced persons and greater demand for humanitarian assistance (Ferris 2010). What these case studies demonstrate is that political instability can weaken governance, social structures, institutions, and coping capacities, by rendering them less capable to respond to or cope with natural hazards/disasters.

## Brief review of complex emergencies

### Conceptualisation of complex emergencies

The phrase, complex emergency, is popular in the humanitarian community and used arbitrarily to describe diverse types and scales of instabilities. Key attributes of complex emergencies recorded across a broad range of crisis settings include that complex emergencies often result from multiple factors associated with war, internal conflict, and terrorism (Abdallah and Burnham 2000); are major humanitarian crises of multi-causal nature requiring a system-wide response; can erode the cultural, civil, political, and economic integrity of established societies (Duffield 1994); result from a combination of political instability, conflict, violence, social inequities, and underlying poverty especially where local and national resources cannot meet the relief needs without international humanitarian assistance (Anderson and Gerber 2018); can erode the cultural, civil, political, and economic stability of societies; and can be exacerbated by diseases further undermining livelihoods and worsening poverty (FAO 2019). Complex emergencies are acute situational crises associated with war and strife resulting to significant fatalities, large population displacement, and shortages of basic human needs (Salama et al. 2004). They describe situations of disrupted livelihoods and threats to life mainly produced by warfare, civil disturbance, and large-scale movements of people, where emergency response is conducted in a difficult political environment (WHO 2020).

The United Nations Office for the Coordination of Humanitarian Affairs (UNOCHA) defines complex emergency as “A humanitarian crisis in a country, region or society where there is total or considerable breakdown of authority resulting from internal or external conflict and which requires an international response that goes beyond the mandate or capacity of any single agency and/or the ongoing United Nations country program”. The key characteristics of complex emergencies highlighted are extensive violence, deaths, population displacement, widespread socio-economic damage, need for large-scale multifaceted humanitarian assistance, political hindrance of humanitarian assistance, and significant security risks for humanitarian workers (UNOCHA 1999).

The various conceptualisations of complex emergencies largely focus on the co-occurrence and interaction between multiple instabilities with a variety of root causes, catalysts, and dynamics where the “complexity” requires the intervention of multiple agencies. This is compatible with the view of complex emergency adopted in this article.

### Complex emergencies in Africa

Complex emergencies have increased considerably in Africa since early 1980s to 112 between 2005 and 2014, affecting around 79 million people (Culver et al. 2017). In fact, complex emergencies were the most frequent disaster forms in sub-Saharan Africa between 2008 and 2018 (USAID 2018). Some took the form of a “protracted crises syndrome^5^”, a “recurring crises syndrome^6^”, and a “contiguum syndrome^7^” (Grunewald 2012). From 1999 to 2015, around 48.5% of all armed conflicts in the world occurred in Africa and the Middle East causing the highest fatalities amongst civilian populations (Pettersson and Wallensteen 2015).

Notable complex emergencies in Africa occurred in South Sudan from 2003 to 2013 due to ethnic clashes and severe droughts affecting 50.6% of the population. Armed violence, floods, and droughts caused complex emergencies in Northern Mali between 2012 and 2013, affecting 50% of the population. Kenya witnessed a complex emergency between 2007 and 2008 as a result of post-electoral violence in Nairobi, resulting in 45,000 to 100,000 refugees. Southern Somalia and Northern Kenya experienced complex emergencies from 2011 to 2012, due to civil conflict exacerbated by floods and droughts (José 2013; Macias 2013). Armed conflict in the Central African Republic from 2012 to 2017 caused 601,000 internally displaced persons and 538,000 refugees. In Ethiopia, civil unrest along the Oromiya–Somali border in 2017 caused 578,000 new internally displaced persons and nearly 884,000 refugees (USAID 2018).

Arguably, the increase of complex emergencies in developing countries can be attributed to resource wars that are driven by social, economic, and ethnic grievances (Duffield 1994), in association with other multi-causal factors such as lingering poverty, climate change effects, changing demographic trends, reversal of development aspirations, and challenges in post-conflict recovery (Macias 2013).

## Conceptual framework for complex emergencies in Africa

The Climate Change and African Political Stability program in the Robert Strauss Centre for International Security and Law has developed a framework on how to identify and distinguish complex emergencies in Africa. The Robert Strauss Centre’s complex emergency framework (Table 1) examines how the various root causes, main consequences, potential responses, and the manifestations of different instabilities (political, economic, demographic, and environmental) combine to form four distinct complex emergencies (acute, chronic, urban, protracted) across Africa. This article uses the Robert Strauss Centre’s complex emergency framework as a benchmark to investigate the Anglophone crisis in order to assess whether the prevailing variables, factors, and consequences of the 4-year-old conflict can be conceptualised as a complex emergency.

**Table 1:** Classification of complex emergencies

Main types of CE	Components	Relative impact assessment	Possible responses	Types of instabilities			
				Political	Environmental	Economic	Demographic
Type 1 Acute	- Acute high-intensity conflict: level is higher than the country’s baseline of violent events - Acute environmental disaster - High level of poverty - Complex social and ethnic geography	- Large affected area - Food insecurity: price hikes - High mortality rates - Concentrated forms of conflict-induced displacement: refugees and IDP settlements - Epidemic outbreaks	**Food aid** - Short-term distribution for displaced persons - Protection of refugees and IDPs **Negotiation** and **coordination** - Open negotiation of a humanitarian access with all the conflict actors - High coordination between the NGOs and agencies - Build resilience	**Acute conflict** Level of violent events higher than the country baseline of recorded events and fatalities	**Environmental disasters** Acute disasters lasting 1 to 15 days (flood, drought, storm, insect infestation, wildfire)	**High level of poverty** High poverty headcount ratio at $1.25 a day (PPP) (% of population)	**Complex ethnic geography** Multiple ethnic groups Demographic pressure
Type 2 Chronic	- Chronic, low intensity of armed and fatal political violence - Vulnerability to climate change-induced hazards - High level of poverty: marginalised region - Changing demographics between groups	- Large affected area - Medium-to-high level of displacement: internal, short term, and circular - Chronic food insecurity: collapse of market and price hikes	**Continued presence in the region and food aid** - Short-term distribution of food aid - Aid to facilitate the resumption of agricultural activities **Long-term measures** - Aid for long-term adaptation to climate change - Plan for integration of conflict parties	**Low-intensity conflict** Low and persistent level of violent events recorded over 5 years	**High level of climate-related hazard exposure** Chronic and long-lasting disasters of over 15 days (drought, floods) with protracted impacts on climate features (rainfall patterns, etc.)	**Marginalised region (vs. rest of the country)** Low GDP per capita under-development of the region	**Settlement in camps** Refugees, IDPs
Type 3 Urban	- High level of civic violence: rioting and protesting - High level of exposure to climate change hazards - High level of unemployment and high percentage of the under-serviced population (public service) - Unstable demographic dynamics: rural-urban migration and urban refugees	- Localised affected area - Epidemic outbreaks - Concentrated forms of displacement - Acute food insecurity: seasonal price hikes - Large slum population	**Better service delivery to population** - Food aid - Education - Vaccination programs - Cooperation over the reinforcement of health institutions **Improve urban governance** - Investment in urban employment - Improved living standards for the poor	**Urban violence** High level of riots and protests in comparison to other types of violence	**High level of climate-related hazard exposure** (rainfall anomalies, chronic water scarcity, cyclones, wildfires, floods, and low-lying coastal zones)	**High level of poverty** High poverty headcount ratio at urban poverty line (% of urban population)	**Migrants in urban areas** **Epidemic outbreak**
Type 4 Protracted	- Absence of central authority and large-scale protracted conflict with multiple non-state actors - Severe vulnerability to climate change induced consistently reoccurring and sudden disasters - High level of poverty and collapse of state and local economies - Disturbed demographics	- Transnational with local hotspots - Epidemic outbreaks - Chronic food insecurity and famine: food availability - Intermittent phases of displacement (e.g. Mogadishu)	**Reinstatement of a central control** **Large-scale poverty reduction programs** - Food aid distribution - Investment for agriculture productivity **Resumption of public services** - Reinforcement of health institutions	**Protracted conflict** Constant high level of violent events and fatalities recorded with periods of over 5 years of acute conflict	**High level of climate-related hazard exposure** Long-lasting disasters of over 15 days (drought, floods)	**Collapse of the national economy** National GDP drop High poverty headcount ratio at $1.25 a day (PPP) (% of population)	**High population of refugees and IDPs** **Collapse of life expectancy**

*Political instability* is defined as that produced by both armed and unarmed conflict directed towards a political target or goal. *Economic instability* is reflected by poverty, vulnerability, and income inequality levels within a population. *Environmental instability* is defined by the occurrence of disasters and long-term shifts due to climate change. *Demographic/health-related instabilities* are observed through urban population growth, complex internal displacement, and epidemics. Source: adapted from Macias (2013)

## Methodology

This is a case study research that is qualitative, exploratory, analytical, and descriptive. Case studies elicit knowledge and understanding of complex social and economic issues (Bowen 2009). The study generated primary data from local, national, international, and civil rights/society organisations working in the Anglophone region—government institutional/organisational reports and briefings; memoranda/reports from opinion, political, religious, and business leaders and the separatist leadership; project plans/programmes and open reports from United Nations organisations and international development agencies like UNICEF, GRID, UNOCHA, WFP, UNHCR, UNAIDS, FAO, IOM, DFID, UKAID, and USAID.^8^

Secondary sources of information included published peer and non-peer review articles, books, manuals, newspaper articles, letters from civil society organisations and opinion leaders from the public/private sector, comments from political activists, technical reports, press releases, survey data in humanitarian databases such as CE-DAT^9^, and information from local, national, and international TV stations.

The documents reviewed for analysis served as produced social facts used to elicit knowledge about the issues under investigation. Content analysis was underpinned by systematically searching, finding, reviewing, appraising, evaluating, synthesising, and triangulating primary and secondary information contained in documents (Krippendorff 2004). The data was examined, interpreted, and categorised into key themes to provide answers to the 1st research objective. Findings on the 2nd objective have been obtained by gauging the consequences of the Anglophone crisis against the components of the Robert Strauss Centre’s framework in order to gain understanding and elicit meaning on determining complex emergencies. The 3rd objective of this research has been achieved by doing a volcanic risk assessment. In-depth analysis of the first three objectives provides insights on conceptualising a new complex emergency paradigm.

Volcanic risk assessment/analysis has three components: hazards, vulnerability, and elements-at-risk characterised by both spatial^10^ and temporal attributes^11^ (Bartolini et al. 2013). In-depth assessment of volcanic eruption hazards involves a process of identifying past volcanic system behaviour in order to infer future behaviour requiring the acquisition of all relevant geophysical information relevant to forecast an imminent eruption scenario (Molist 2017). Nevertheless, a detailed scientific volcanic eruption risk assessment is beyond the scope of this article. The Cameroon government’s funded GRINP (*Gestion des Risques Naturels et Protection Civile* or Natural Risk Management and Civil Protection) project that assessed volcanic hazard risks on Mt. Cameroon (Thierry et al. 2008) has informed the risk assessment in this article. The motive is to identify the eruption frequency in order to determine the available time window prior to the next eruption (Molist 2017).

This approach is based on the premise that volcanic systems have characteristic eruption recurrences and active volcanoes with high eruption frequencies can be more easily forecast than those with lower eruption frequencies (Scandone et al. 2016). This underpins the volcanic risk assessment approach adopted that focuses on the eruption frequency on Mt. Cameroon. Nevertheless, there is uncertainty to determine the exact time and behaviour of a volcanic eruption (Molist 2017).

## Research results—ramifications of the Anglophone crisis

### Armed conflict

Attempts by government security forces to vigorously quell down peaceful protest have been blamed for the insurgence of several armed militias in the Anglophone region (UNOCHA 2018). The armed groups have a secessionist agenda and have been in confrontation with government forces in a conflict dubbed the Southern Cameroons (Ambazonia) war of independence. Anglophone secessionist groups are demanding an independent Ambazonia Republic (GRID 2019). The main militias operating in the region are the Ambazonia defence forces, Southern Cameroons Defence Forces, Southern Cameroons Restoration Forces, Red Dragons, Bafut Seven Karta, Manyu Ghost Warriors, Amberland Forces, Amberland Quifos, Amberland Marine Forces, Manyu Ghost Warriors, Menchum Falls Warriors, Tigers of Ambazonia, Warriors of Nso, White Tigers, and Vipers. The number of fighters in each of these groups is estimated to range from around a dozen to more than 500 (ICG 2019). The militias have allegiance to different Cameroon diaspora organisations purportedly sponsoring them. Notably are the Southern Cameroons Liberation Council^12^, the Ambazonia Governing Council, and the Ambazonia Defence Council (ICG 2019; AFP 2018; JournalduCameroun 2020).

To expedite military victory, the government increased its fire power by deploying helicopters and armoured vehicles and numerically reinforced its elite special forces. Despite these efforts and casualties suffered by the militias, they still control vast rural areas and main roads in the conflict region. The conflict has caused over 3000 fatalities and affected around 1.3 million people (internally displaced persons and refugees included) with dreadful repercussions for the population of the region (APF 2018; Craig 2020).

### Insecurity and lack of authority

**Fig. 2:**
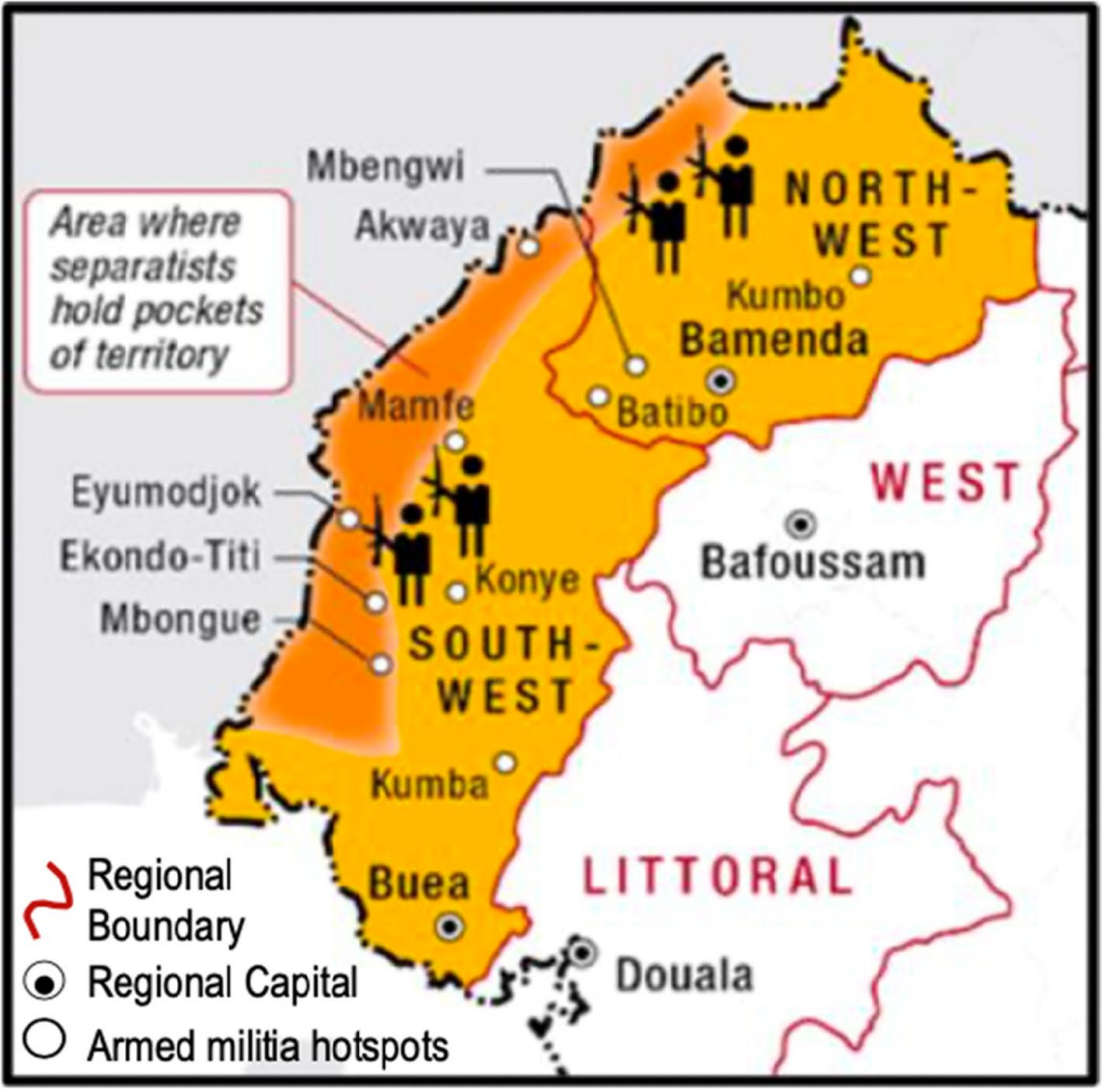
Map of the Anglophone region showing pockets of territory allegedly controlled by the separatists. Source: adapted from the ICG (2019)

The government’s loss of full control of the region creates an environment of insecurity for the population, humanitarian space, and the belligerent forces. There is power tussle between the government and militia/secessionist groups to control geographical areas. The Interim Government of the Republic of Ambazonia^13^, the Ambazonia Governing Council, and some political activists in the diaspora are using the militia forces and secessionist groups to control huge areas in the restive region (Fig. 2). The terrified population has respected orders to boycott schools and violate administrative orders. Separatist fighters are alleged to have attacked students, teachers, and the population to enforce school boycott orders. Furthermore, they have been accused of kidnapping denizens and relief workers for ransom; hence, non-governmental organisations have limited their operations. Government security forces are accused of raiding schools, religious premises, and hospitals hosting internally displaced persons and indiscriminately killing civilians and burning houses/markets in separatist strongholds. In order to curb the violence, the government has taken security measures such as imposing curfews; restrictions on gatherings, movements of people, and circulation of cars/motorbikes; and including more checkpoints (Craig 2020; ICG 2019; UNOCHA 2018; Nsonzefe 2019; JournalduCameroun 2018a).

The traumatised population has been caught between government and separatist forces. Communities in areas where the armed groups operate dread the presence of security forces, alleged to kill indiscreetly in communities hosting separatist fighters. According to Lazar (2019), the levels of distrust in the security forces have increased sharply in the Anglophone regions since 2015 from 22 to 62%. Likewise, communities perceived to be in support of the security forces are at the mercy of the militia groups.

### Human rights violations

The Anglophone crisis is characterised by human rights violations. On January 17, 2017, the government shut down Internet access in the restive region for 93 days to curtail dissent amongst the population. This was regarded by many local and international organisations^14^ as an outright violation of constitutional and human rights to access information and freedom of expression (Calis 2018).

Government security forces have been accused of using excessive force, extrajudicial killings, torture, and ill-treatment of suspected separatists and detainees. They have also razed and burnt property including houses in more than 170 villages in the Anglophone region. A notable case is the alleged massacre of 21 unarmed civilians in Ngarbuh village (North West Region) on February 14, 2020.^15^ After this tragic incident, government officials accused some aid agencies of colluding with the separatist to incriminate the security forces.^16^ In June 2019, the government started to systematically track and vet humanitarian operations in the region.^17^ Accusations have also been levied against armed separatists for abductions and killing of security forces, administrators, and civilians perceived to be colluding with the government (Ajumane 2018; Craig 2020; Egeland 2019; HRW 2018, 2020; GRID 2019; ICG 2019).

Contemporary data on political freedom in the crisis region is staggering. An estimated 81% of Anglophones have less freedom to express their political views than a few years earlier and they are twice more likely to fear violence and political intimidation than Francophones (Lazar 2019). The restive region massively boycotted the October 2018 presidential elections and the February 2020 Municipal and Legislative elections that came on the heels of a national dialogue to resolve the Anglophone crisis. The national dialogue is considered by some critics a failure since fighting continues unabated.

### Massive population displacement

**Fig. 3:**
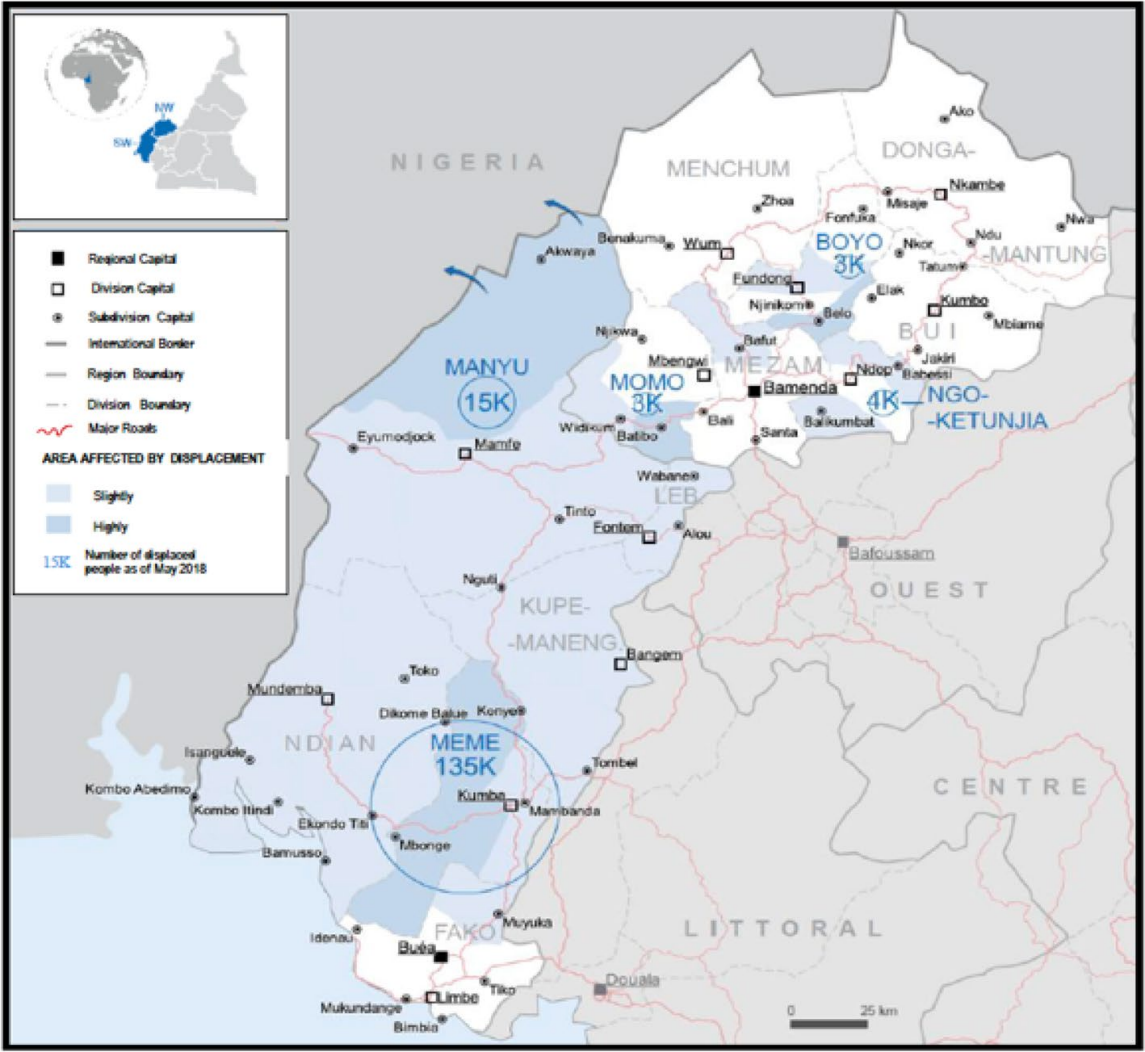
Map of the North West Region and South West Region showing areas most affected by population displacement. Source: adapted from UNOCHA (2018)

The Anglophone crisis has caused over 900,000 internally displaced persons and 60,000 refugees (Craig 2020; ICG 2019). Thousands of people have fled to the predominantly French-speaking region and across the border into Nigeria. In villages that are conflict hotspots, around 80% of the inhabitants have escaped and sought refuge in the bushes/forest. The most hit areas are Boyo and Meme Divisions of the North West Region and the South West Region respectively (Fig. 3) where dozens of villagers have almost been emptied of their population (UNOCHA 2018; UNHCR 2019; GRID 2019).

### Consequences on public health

The conflict is having a serious toll on the health of the population. Internally displaced persons and the populace that fled to the bushes are living in unsanitary and unhygienic conditions. There is rampant open-air defecation enabling the possible spread of diseases in the crowded living conditions in the bushes. The most vulnerable are the elderly, pregnant/lactating women, young girls out of school, and children under the age of 5.

The insecurity has caused the closure of many health facilities. Many hospitals and health centres (especially in the rural areas) have been attacked and torched. Others have been abandoned because their staff are afraid of being kidnapped or caught in the crossfire (UNOCHA 2018; Egeland 2019). As of December 2018, an estimated 34% of health facilities in both regions were not functioning (Craig 2020). Those left are often poorly equipped and under pressure to treat the influx of the sick. Limited access to adequate health care exacerbates the suffering of the Anglophone population.

The COVID-19 pandemic has exposed the fragile state of Cameroon’s health system (Bang et al. 2020) and further compounded the health problems of the population in the Anglophone region. The website of Cameroon’s Ministry of Public Health reported 21,430 cases of coronavirus on October 22, 2020, with just 424 deaths. This figure is probably underreported since COVID-19 testing and data collation has not fully included insecure areas due to the Anglophone crisis and the Boko Haram insurgency.

We contend that while the pandemic can exacerbate the Anglophone crisis, the crisis can also affect the virus spread. The conflict restricted movement in the Anglophone region. This did not change much with COVID-19. The limitation on movement and lockdowns can prevent transmission of the virus. On the other hand, the restrictive measures imposed by the government to control the virus increased the suffering of the populace. A report about COVID-19 quotes a man in the North West Region who described both the Anglophone crisis and COVID-19 as “monsters” that have seriously affected his ability to feed his family despite being jobless (Song 2020). The OCHA (2020) estimated that only 46% of the population in the conflict-ridden Anglophone regions have access to information required for the prevention of COVID-19. This has implications for implementing infection prevention/control measures disseminated to the public. With 34% of the health facilities in the conflict-ridden region not functioning (Craig 2020), access to medical care for COVID-19 is limited.

### Consequences on education

Education has been at the core of the dissenting voices of the Anglophone problem. All the seven legalised Anglophone teachers’ trade unions^18^ launched a campaign to abolish the unfair treatment of the English/Anglo-Saxon education sub-system in Cameroon. The conflict has repercussions for education due to separatist demands for educational establishments in the region to shut down until all education grievances that led to the crisis have been resolved. Educational facilities that defy the orders have been subjected to violence. Schools have been destroyed and their teachers/students threatened, abducted, and/or killed. In response, most educational institutions have been abandoned, keeping over 780,000 children out of school. Indeed, over 80% of schools have been closed in the restive region (UNICEF 2019). Consequently, primary school to university attendance dropped drastically with implications for enrolment to write the General Certificate of Education Ordinary and Advanced Levels.^19^ In 2017, enrolment for the General Certificate of Education Ordinary and Advanced Levels dropped by 28% increasing to 75% in 2019—some students have relocated to study in the crisis-free regions. The domino effect of non-school attendance is social ills like child exploitation and child labour, early marriages, teenage pregnancies, juvenile delinquencies, increased teenage alcohol consumption and drug use, and more stealing/robbery (UNOCHA 2018; Egeland 2019; JournalduCameroun 2019a; ICG 2019: Craig 2020; Ludovica 2016; UNICEF 2019). These issues have long-term implications for increased illiteracy and poverty.

University education has also been affected in the region’s two Anglo-Saxon universities. Insecurity has kept some students away from the campuses and constantly disrupts academic activities. University lecturers are vulnerable to attacks and kidnapping. Many are frightened to continue working normally and have been seeking to leave the country. In 2019, the Institute for International Education Scholar Rescue Fund in the USA received an unusually high number of applications from Anglophone Cameroon scholars citing threats to their lives and academic carriers amidst the ongoing Anglophone crisis (IIE 2020).

### Economic impact/effects

The Anglophone crisis is having huge economic and financial repercussions. Cameroon’s Gross Domestic Product growth rate, which was 5.8 in 2015 prior to the crisis, was downgraded to 3.9 in 2019 (World Bank 2017; IMF 2019). The Internet shutdown seriously affected Cameroon’s economic growth with an estimated loss of Franc CFA 499 billion (US$ 846 million) (Mboumien 2018). Weekly “ghost towns”, restrictions on movement^20^, and insecurity are affecting business transactions through slowed flow of goods, people, and services. Businesses and transport vehicles that refuse to respect “ghost town” days have been threatened and targeted. The security forces have also been accused of vandalising and burning business premises and markets. Consequently, hundreds of businesses have been paralysed in the region.

Insecurity has forced production and operational activities of the biggest companies in the region like the Cameroon Tea Estate^21^, the Upper Nun Valley Development Authority^22^, and the Cameroon Development Corporation^23^ to dwindle. Exports of the major products from the region like banana, palm oil, coffee, and rice production dropped dramatically. Indeed, the Cameroon Development Corporation reduced its operational capacity to 26%^24^ and recorded a net loss of Franc CFA 32 billion (around $55.3 million) during the 2018 fiscal year (Mbodiam 2020; Kindzeka 2019). This has huge implication for employment since the Cameroon Development Cooperation (located on the slopes of Mount Cameroon) is Cameroon’s second largest employer that employs people from all regions of the country.^25^

Unemployment has soared in the region with over 30,000 more people rendered jobless due to the crisis. Cameroon’s Employers’ Association estimated that the formal economy of the region had lost around 6434 jobs by July 2018 and a further 8000 jobs were at risk. The unemployment rate in Buea—the regional capital of the South West Region, for instance—rose to 70%. The North West Region’s custom department reported revenue losses of over Franc CFA 200 million (around $362,000) for the 2018 fiscal year and the overall losses to Cameroon’s economy were estimated at Franc CFA 269 billion (around $489 million) for the same period. Road contractors have been attacked and threatened and their equipment vandalised/destroyed causing many to stop/abandon projects. Indeed, more than 80% of the public contracts awarded in the Anglophone region for the 2017/2018 fiscal year were not executed (ICG 2019; Mbodiam 2020; APF 2018).

While the economic effects are gloomy for Cameroon, some individuals, interest groups, and a few institutions have benefited. Business is probably booming for contractors supplying arms to the security forces and separatist fighters. Some political jobs were created and filled by Anglophones for the first time in Cameroon’s history—Anglophones were appointed as Ministers of Territorial Administration and Secondary Education. The government created a commission for the promotion of bilingualism and multiculturalism and a national disarmament, demobilisation, and reintegration committee with Anglophones at their helm. Furthermore, common law divisions were created at Cameroon’s supreme court and the National School of Administration and Magistracy (Nsom 2017; Republic of Cameroon 2017). These appointments and new institutions were in a bit to diffuse the crisis.

### Consequences on culture

The rich Anglophone culture has not been left unaffected. Traditional rulers and custodians of the cultures and traditions of the Anglophone region (particularly those perceived to be collaborating with the government) have been publicly attacked, beaten, kidnapped, or killed. In February 2018, armed men killed the supreme chief of the Balondo people in Ekondo Titi (South West Region). A few months later, 8 chiefs in Fako Division were kidnapped.^26^ Inevitably, dozens of tribal chiefs went into hiding and currently live out of their palaces (JournalduCameroun 2018b; Kindzeka 2018).

In the North West Region, women have carried out burial rites in some of the crisis hotspots—a role traditionally reserved for men. Women were forced to bury corpses in Belo village (Boyo Division) in July 2018, when all the boys/men had fled from the security forces. Furthermore, dramatic footages emerged from the press and social media in January/February 2019 showing women carrying out burial rights in Bafut village (Mezam Division) after a military raid on armed separatist groups caused all the men to flee from the village (Journalducameroun 2019b). Prior to the crisis, it was a taboo in the cultures/traditions of the Anglophone region for women to perform burial and funeral rights.

Furthermore, palaces have been attacked and looted. For instance, in September 2018, Cameroon’s Rapid Intervention Battalion soldiers damaged parts of the roof of the Royal Palace in Bafut (North West Region) with the pretext of searching for separatist fighters. Under the same guise, soldiers raided the same place on 24 September 2019 and almost killed the brother of the paramount Fon of Bafut (Abumbi II) when he was shot and wounded. They then allegedly looted the museum in the palace and took several rare centuries-old artefacts. In both instances, no separatist fighters were found (HRW 2019). Ironically, under the recommendation of Cameroon’s Ministry of Arts and Culture, UNESCO had placed the Royal Bafut Place on its tentative List of World Heritage Sites (UNESCO 2018). The government, therefore, has the obligation to protect the Bafut Royal Palace and preserve its artefacts, since the palace is one of the most significant historical and cultural heritage sites in the country.

### Humanitarian assistance

The conflict has triggered the request for urgent funding needed to meet massive humanitarian needs (UNICEF 2019). Many humanitarian organisations have done needs assessments in the region in diverse areas: food security (FAO, WFP); shelter and non-food items (UNHCR); protection (IOM, IMC, UNFPA, IMC, UNICEF, UN Women); health (AHA, IMC, UNAID, UNFPA, WHO, UNICEF); education (UNICEF); water, sanitation, and hygiene (UNICEF, PIC, UNDSS, OCHA); safety (UNDSS); and coordination (OCHA) (Egeland 2019; UNOCHA 2018). UNICEF has so far supported more than 140,000 children; provided 30,000 children with psychological support identified 972 displaced and unaccompanied children and plans to reunite them with their families; and supplied water, sanitation, and hygiene kits to over 78,000 people (UNICEF 2019). Despite the zeal to help, the humanitarian organisations were able to reach just 40% in dire need of assistance in 2019 and only 18% of the requested budget was funded (Craig 2020). This implies the operation is suboptimal. As the crisis persists, the humanitarian and funding needs will further increase, limiting the capacity of the aid agencies to deliver.

## Ascertaining whether the Anglophone crisis is a complex emergency

This section compares the attributes of the Robert Strauss Centre’s complex emergency framework (Table 1) with the ramifications of the Anglophone crisis to determine whether the conflict is a complex emergency. The assessment has been done using a Likert scale—low relevance (value 1) to extremely relevant (value 4) (Table 2). Assessment of the ranking is based on how close the variables in the Robert Strauss Centre’s complex emergency framework conform with findings on the ramifications of the Anglophone crisis. In doing so, we are aware that the variables depicting the diverse types of complex emergencies cannot exactly align with the effects of the Anglophone crisis since no two complex emergencies in the world can exactly be the same (World Vision 2018). Therefore, the following assumptions were made: (1) the criterion for environmental/climate change-induced hazards/disasters in the framework has been rated as “low relevance” since the Anglophone crisis is not due to environmental/climate change; (2) when considering “Type of Instability”, the number of years of conflict ‘five’ mentioned against political instability is not a key factor considering the Anglophone crisis has not reached 5 years when this paper was written; (3) the “slum” population mentioned against “Urban complex emergency” is likened to the displaced population living in the bushes.

**Table 2:** Comparism of the Robert Strauss Centre’s complex emergency framework with findings from the Anglophone crisis to determine whether the latter is a complex emergency

Robert Strauss Centre’s complex emergency framework			Relevance to the Anglophone crisis/scores				Scale score	
Complex emergency types and themes		Variables	Low relevance	Relevant	Very relevant	Extremely relevant	Theme scores	Types of complex emergency scores
			1	2	3	4		
**Acute**	**Components**	Acute high-intensity conflict: level is higher than the country’s baseline of violent events				✓	10	36
		Acute environmental disaster	✓					
		High level of poverty				✓		
		Complex social and ethnic geography	✓					
	**Relative impact assessment**	Large area affected				✓	15	
		Food insecurity price hikes			✓			
		High mortality rates			✓			
		Concentrated forms of conflict-induced refugees and internally displaced persons				✓		
		Epidemic outbreaks	✓					
	**Types of instability**	**Political (acute conflict):** level of violent events higher than the country baseline of recorded events and fatalities				✓	11	
		**Environmental (environmental disasters):** acute disasters lasting 1 to 15 days (flood, drought, storm, insect infestation, wildfire)	✓					
		**Economic (high level of poverty):** high poverty (HP) headcount ratio at $1.25 a day (PPP) (% of population)			✓			
		**Demographic (complex ethnic geography):** multiple ethnic groups, demographic pressure			✓			
**Chronic**	**Components**	Chronic, low intensity of armed and fatal political violence			✓		11	29
		Vulnerability to climate change-induced hazards	✓					
		High level of poverty: marginalised region				✓		
		Changing demographics between groups			✓			
	**Relative impact assessment**	Large area affected				✓	8	
		Medium-to-high level of displacement: internal, short term, and circular		✓				
		Chronic food insecurity: collapse of market and price hikes		✓				
	**Types of instability**	P**olitical (low-intensity conflict):** low and persistent level of violent events recorded over 5 years		✓			10	
		**Environmental (climate-related hazard exposure):** chronic and long-lasting disasters of over 15 days (drought, floods)	✓					
		**Economic (marginalised region vs. rest of the country)** Low GDP per capita under-development of the region			✓			
		**Demographic (settlement in camps)**: refugees, internally displaced persons				✓		
**Urban**	**Components**	High level of civic violence: rioting and protesting			✓		11	30
		High level of exposure to climate change hazards	✓✪					
		High level of unemployment and high percentage of the under-serviced population				✓		
		Unstable demographic dynamics: rural-urban migration and urban refugees			✓			
	**Relative impact assessment**	Localised affected area	✓				9	
		Epidemic outbreaks	✓					
		Concentrated forms of displacement			✓			
		Acute food insecurity: seasonal price hikes		✓				
		Large slum population		✓				
	**Types of instability**	**Political (urban violence):** high level of riots and protests compared to other violence			✓		10	
		**Environmental (high level of climate-related hazard exposure):** rainfall, chronic water scarcity, cyclones, wildfires, floods, and coastal zones	✓					
		**Economic (high poverty):** urban poverty headcount ratio (% of urban population)			✓			
		Demographic (migrants in urban areas)			✓			
**Protracted**	**Components**	High level of unemployment and high percentage of the under-serviced population			✓		16	34
		Unstable demographic dynamics: rural-urban migration and urban refugees			✓			
		Absence of central authority and protracted conflict with multiple non-state actors			✓			
		Severe vulnerability to climate change-induced reoccurring and sudden disasters	✓					
		High level of poverty and collapse of state and local economies			✓			
		Disturbed demographics			✓			
	**Relative impact assessment**	Transnational with local hotspots	✓				6	
		Epidemic outbreaks	✓					
		Chronic food insecurity and famine: food availability		✓				
		Intermittent phases of displacement		✓				
	**Types of instability**	**Political (protracted conflict):** constant high level of violent events and fatalities recorded with periods of over 5 years of acute conflict				✓	12	
		**Environmental (high level of climate-related hazard exposure):** long-lasting disasters of over 15 days (drought, floods)	✓					
		**Economic (collapse of the national economy):** national GDP drop, high poverty headcount ratio at $1.25 a day (PPP) (% of population)			✓			
		**Demographic (high population of refugees and internally displaced persons):** collapse of life expectancy				✓		
**Total scores**			14	7	19	11		
**Percentage of scores**			27.5	13.5	37.5	21.5		

The assessment shows that the Anglophone crisis is a complex emergency because 72.5% of the variables in all the types of complex emergency fall within the relevant to extremely relevant ranking criteria. The most probable type of complex emergency is the acute complex emergency since this has the highest score (36) compared to the other types of complex emergencies. On this basis, we conclude that the Anglophone crisis is arguably an acute complex emergency. While acknowledging this analysis is subjective, it paints an incredibly good overall picture of the Anglophone crisis, as seen through the lens of the Robert Strauss Centre’s complex emergency framework.

## Probability of an imminent Mt. Cameroon eruption

A key attribute of Mt. Cameroon as mentioned earlier is the high frequency of eruptions. Seven major eruptions have occurred in the last 120 years—1909, 1922, 1954, 1959, 1982, 1989, and 1999. The 2000 eruption was considered a continuation of the 1999 eruption. The eruptions last on average for 3 weeks with each releasing a volume of around 5 × 10^6^ m^3^ to 6.6 × 10^7^ m^3^ of lava (Suh et al. 2003). Basic statistical analysis reveals an average of around 18-year interval between eruptions. The closest variation of years between eruptions ranges from 17 to 23 years, extending to 32 years. Considering the last major eruption was in 1999/2000, we posit that Mt. Cameroon is already 2 years into its window of eruption, which could extend to 2032 or beyond. This implies there could be a major eruption at any moment.

**Fig. 4:**
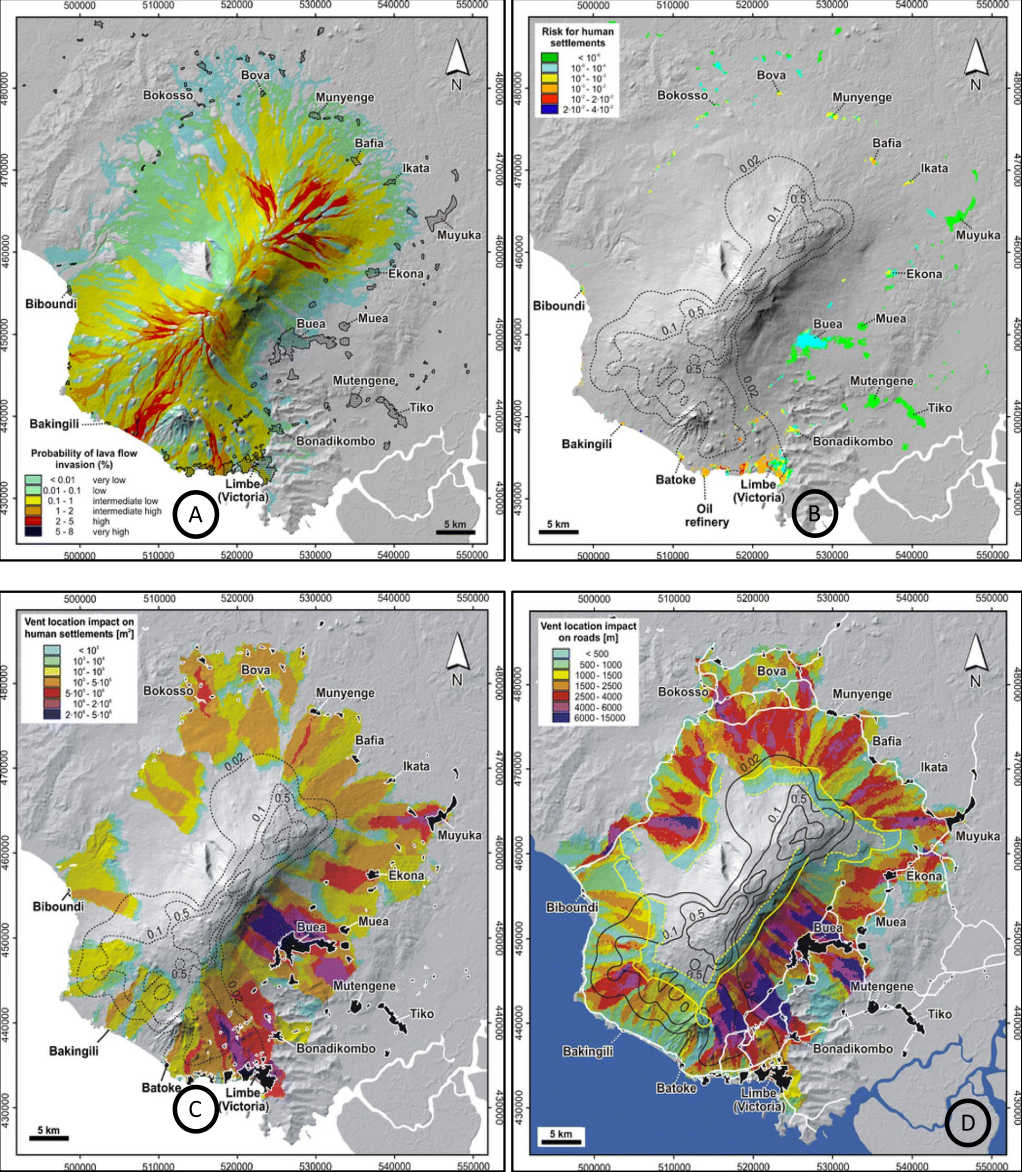
Risk maps of probability of lava flow inundation (**A**), human settlements (**B**), vent location impact on human settlements (**C**), and vent location impact on roads (**D**) on Mt. Cameroon. Source: Favalli et al. (2012)

The eruption could be associated with earthquakes and be explosive and could produce voluminous lava flows. These hazards would seriously threaten nearby population centres and villages (Tsafack et al. 2009) that are hotspots of the Anglophone crisis. Risk maps of the region show the probability of lava flow inundation (Fig. 4A), lava flow risk for human settlements (Fig. 4B), and an overview of the expected threat (impact, damage) of vent location leading to lava flow on the surrounding towns/villages (Fig. 4C) while Fig. 4D shows the potential damage of roads per kilometre of lava flow.

Notable is the high vulnerability of big towns like Buea (90,000 people) and Limbe (85,000 people), including villages like Muea, Ekona, Muyuka, Mutengene, and Tiko (Bonne et al. 2008; Wantim et al. 2013). The fertile volcanic soils of these towns and their surroundings also host plantations of the Cameroon Development Cooperation. A major eruption would seriously threaten agricultural activities on the plantations. Indeed, any major damage of commercial export crops on the plantations by lava flow, as has been the case in the past, or disruption of farming activities, may have huge cascading effects that could stifle Cameroon’s economy. In addition, that would cause more population displacement, threaten infrastructural development, and aggravate response to the Anglophone crisis and COVID-19, thereby creating a higher level of emergency.

## A new paradigm—the “Complex Disaster Emergency” (CDE)

**Fig. 5:**
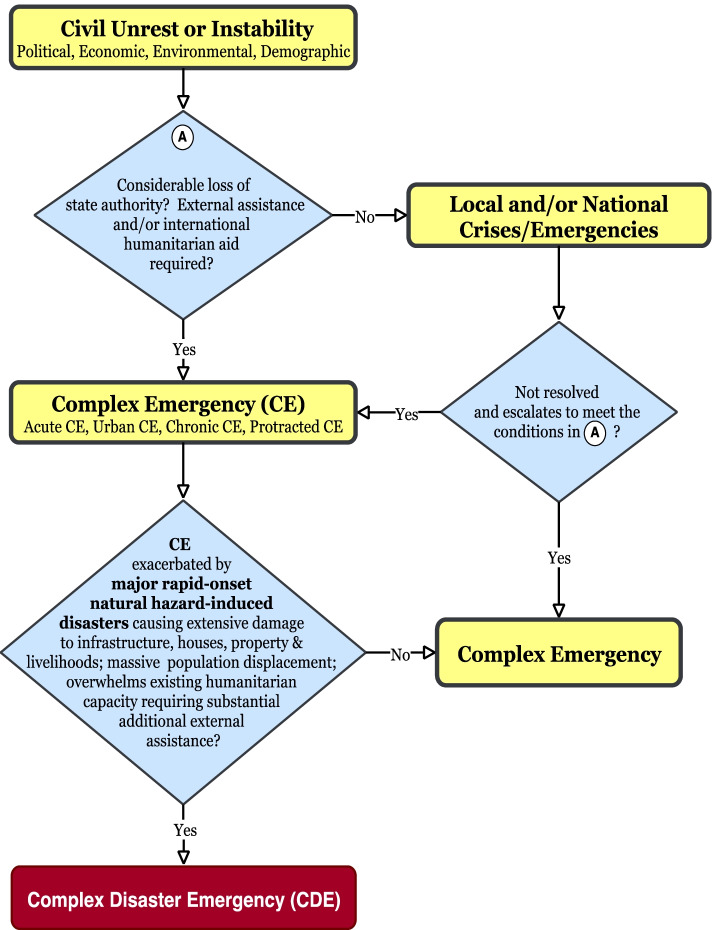
New conceptual framework for CDE. Source: authors

We are suggesting a new complex emergency paradigm, the “Complex Disaster Emergency” (CDE) that could arise if a Mt. Cameroon eruption overlaps with the established acute complex emergency. In complex emergencies, there is a tendency to overlook the risk of other hazards that could interfere or worsen the challenges of dealing with the existing crisis. Already extremely stretched by multiple humanitarian crises including the COVID-19 pandemic, the prospect of the Cameroon government and humanitarian actors to deal with multiple or dual intersecting crises in the Anglophone region is daunting. The analysis in the previous section has revealed the high likelihood of a volcanic eruption on Mt. Cameroon in the nearest future. We posit that any major eruption that overlaps with the ongoing acute complex emergency would lead to more population displacement, fatalities, damage to infrastructural developments, and livelihood assets. Inevitably, more suffering, destitution, and poverty will ensure. Such concurrent events would overwhelm the humanitarian response requiring even more humanitarian assistance. In short, the “complexity” of the existential Anglophone crisis would increase, escalating the established acute complex emergency to a higher level conceptualised as CDE. Figure 5 is a flow diagram showing this process.

This article serves as an early warning for a potential CDE in the South West Region that could further complicate the existential acute complex emergency. Considering only 40% of the humanitarian assistance needed in the region has been provided (Craig 2020), a CDE would completely swarm the local, national, and international response with dire repercussions to the destitute population.

## Discussion

Arguably, the Anglophone crisis was inevitable due to the incumbent government’s negligence in adequately identifying, monitoring, managing, or responding to emerging socio-economic and political risks for several decades. The reluctance of the government to rapidly resolve legal and educational grievances in the Anglophone region peacefully and tactfully seem to have transformed it into an acute complex emergency. For over 4 years now, violence has become an indispensable adjunct of socio-economic, cultural, and political endurance creating a tense living environment where insecurity thrives.

Despite the escalating conflict and its growing longevity, the government has been intransigent to separatist requests to discuss the form of the state (independence from French Cameroon) in the presence of an international mediator. The government continues to predominantly use military means to resolve the crisis and separatists seem to have vowed to continue fighting for independence. The national dialogue held in October 2019 to end the crisis has not yielded much fruits. Rather, the fighting and mayhem persists. Those actually benefiting are probably the private for-profit organisations supplying arms to the belligerent forces.

The conflict is becoming more complex, entrenched, and likely to be protracted since many actors are involved.^27^ The conflict may escalate into a full-blown revolutionary war if many Anglophones loose fundamental trust in the state. Ending the crisis requires more inclusive dialogue between the government and the separatist leadership—incarcerated in August 2019. More international pressure is needed for the opposing sides to make concessions in order to reverse the spiral of violence and eventually stop the conflict.

This article has revealed the conspicuous horrendous consequences of the crisis. The loss of livelihoods and huge displacement of people are exacerbating hunger and poverty especially for the most vulnerable. The dominantly agrarian livelihoods in the region have been seriously affected. Meeting the basic needs of the population like having at least two meals per day is a daunting prospect for the vulnerable population. In consequence, inflation has been soaring due to dwindling outputs and regular interruptions in the supply chain of agricultural products and manufactured goods from other regions of the country.

While there is much attention to the short-term impacts of the conflict, the cascading effects may cause a systemic crisis. Warfare and insecurity continue to disrupt education, communication, transportation, movement of goods/services, and cashflow. This poses a grave threat to the country’s economy that has already lost trillions of Franc CFA. The long-term consequences on education will also be far reaching. For instance, the credibility of the General Certificate of Education results from 2019 going forward, especially for candidates in the troubled regions, leaves much to be desired owing to ineffective studies since the sitting Ordinary Level candidates were in form 3 when the crisis started. As for the Advanced Level students, they entered high school in September 2017 when the crisis had affected the region for almost a year.

Since the Anglophone crisis is occurring against a backdrop of other humanitarian emergencies, there is additional pressure on the humanitarian stakeholders to deal with the multiple conflicts. The diversity and amount of relief needed continuous to soar tremendously. The impact on critical infrastructure and disruption of trade with implications for the smooth functioning of the markets is bound to escalate the needs as well. It is therefore not surprising that the humanitarian actors on the scene are struggling to cope with the speed and rising scale of destitution, hunger, and displacement.

Non-governmental organisations operating in the conflict zone have rightfully condemned human rights violations. But the government has reacted furiously when cautioned against the excessive use of force on innocent civilians and threatened to ostracise non-governmental organisations that speak against such activities. Governments’ overreaction is jeopardising the decent work of the non-governmental organisations with implications for the survival of the affected population. The government should increase its effort to document and bring to book those accused of human rights abuses.

A key outcome of this article is the imminent eruption of Mt. Cameroon that could dramatically escalate the Anglophone crisis (established in this article as an acute complex emergency), into a CDE. Consequently, having knowledge and understanding of the longer-term dynamics of the acute complex emergency and how it can be transformed into a CDE is critical. Considering that complex emergencies cause enormous human suffering and toll, the priority is to save lives by providing protection, security, and essential relief/needs to the affected population (Anderson and Gerber 2018). In June 2019, the Anglophone crisis topped the list of the most neglected crises in the world (Egeland 2019) despite the increasing humanitarian needs and insecurity that has weakened the socio-economic pillars of the region. This is concerning since the provision of needs has, so far, elicited extremely limited relief response with aid reaching less than 40% of the beneficiaries (Craig 2020). Therefore, the prospect of responding to a CDE would be harrowing considering needs assessment and relief operations would require bigger and more complicated operations.

The Anglophone crisis has compounded pre-existing vulnerabilities and has substantially eroded the long-term livelihoods and coping strategies of the affected population, including the local asset/resource base of the region and country. Consequently, the crisis has been exacerbating poverty and hardship in the region. The chaos that could arise from a major eruption would cause more population displacement that could significantly degrade the human, social, and financial capital of the region and requiring much more funding to stabilise the situation.

Ultimately, a CDE would require more foreign assistance or humanitarian aid. Whether sufficient external assistance can be available or not, the state has the primary responsibility for mitigating disaster risks including protecting and responding to crises (UNDRR 2015). Considering that Cameroon’s disaster framework is struggling to manage contemporary emergencies/disasters in the country (Bang et al. 2019a), responding to a CDE would be very challenging. Trying to reduce vulnerabilities and build resilience among the communities facing a CDE will be an uphill task if adequate contingency plans are not taken. Hence, the government and its international partners need to engage in a risk reduction and preparedness activities in anticipation of a CDE.

## Recommendations

First, timely and effective risk assessment (to determine additional resource needs) in preparation for a potential CDE is needed to identify the underlying patterns of vulnerability and enhance the resilience of the affected population. Second, contingency planning for effective crisis communication is a critical consideration especially in Buea where strategic/critical institutions essential to DM are located. Having appropriate communication strategies to sensitise the population is essential. Third, security will be fundamental for any response operation to be successful. In view of the ongoing conflict, it will be vital to set up security corridors in case of urgent evacuation. Forth, contingency evacuation plans should be set up that identify the poorest and most marginalised communities that would be prioritised for relocation, relief, and rehabilitation purposes. Fifth, preparedness to implement mitigating interventions requires a conducive funding environment. More funds will have to be allocated in preparation for a CDE. This remains questionable, given that most funding countries are currently diverting huge resources to combating the COVID-19 virus. Sixth, like funding, issues with interagency coordination/cooperation have been undermining the effective performance of Cameroon’s DM system (Bang et al. 2019a). The role of Cameroon’s Directorate of Civil Protection to coordinate DM activities should be reinforced at a higher governmental level to avoid confusion in a CDE crisis situation. These activities may be challenging in a hostile and insecure environment where the population of the region demonstrates diminishing trust in the government. The inability to enforce security, control communication/transportation channels, and gain the trust of the conflict-ridden population could further impede the response abilities of the government.

While it is inevitable that a heightened crisis could lead to overreliance on external humanitarian assistance, there is no guarantee that foreign agencies would be swift in their response to a CDE scenario. That is partly because the international aid apparatus is often lopsided and has been designed to function in a complex emergency, though strained in many situations as this study has shown. Nevertheless, preparedness may be strong amongst international aid agencies in terms of skills and experience. But as long as there are uneven and divided power relations in the conflict area, it may be difficult for aid agencies to access donor funding. In operational terms, a CDE could make civilian tracking and accessing more difficult, rendering data collection and analysis relevant for sourcing and tracking humanitarian relief exceedingly difficult. Therefore, a CDE would be more demanding to manage and those to suffer most are vulnerable groups like children, the elderly, the disabled, and women. Any interference in the work of non-governmental organisations may weaken their ability to do in-depth vulnerability assessments, even when financial resources were available. Therefore, sustained and strong advocacy would also be relevant to create an enabling climate to provide life-saving assistance in accordance with humanitarian principles and international humanitarian law (UNOCHA 2018). For the international humanitarian community also, strategic contingency plans that articulate a stronger coordination, collaboration, crisis communication, and increased accountability with other stakeholders would be needed. Such plans should be pretested between internal and external stakeholders so that all parties can have a common situational awareness in terms of the likely operational environment of a CDE.

## Conclusion

In 2016, valid grievances by the lawyers’ and teachers’ trade unions in Cameroon’s Anglophone regions urging the government to revamp the Anglophone legal and education systems quickly degenerated into an armed political crisis due to government’s intransigence. The crisis has had dire socio-cultural, economic, and political consequences. This research has revealed the following ramifications of the Anglophone crisis: armed conflict between several secessionist militia groups and government security forces resulting to thousands of fatalities, massive population displacement leading to internally displaced persons and refugees, and violence, insecurity, and human rights violations. The conflict has also dealt a massive blow to the economy, health, and culture of the two Anglophone regions. The ramifications of the crisis were assessed against the Robert Strauss Centre’s complex emergency framework and determined to be an acute complex emergency. Historical risk analysis of Mt. Cameroon’s eruption hazard has confirmed that we are presently in the time window of an eruption that could happen at any moment. We have posited that a major eruption would dramatically escalate the crisis to an astronomical level, conceptualised as CDE.

This article has argued that there is a need for humanitarian stakeholders to strengthen preparedness for complex emergencies that can be exasperated by natural hazards. Resource-scarce developing countries tend to be reactive to disasters, crisis, and emergencies while the humanitarian discourse has become overly focused on complex emergencies. These priorities are at the expense of understanding the dynamics and complexities involved in responding to a complex emergency that could be exacerbated by a major natural hazard or CDE. We have also argued that government and humanitarian agencies need to have foresight of mega crises that may result from the interaction of human-induced and natural hazard (disaster) incidents occurring concurrently. A number of recommendations have been suggested to that effect.

The recommendations challenge Cameroon’s DM frameworks and both internal and external humanitarian actors to embrace and expand their knowledge base on potentially more complex humanitarian crises. Transforming such knowledge into timely risk assessment would better prepare them for a CDE scenario. However, being proactive to prevent human-induced conflict is paramount. Even when mistakes have been made and civil unrest has metamorphosed into armed conflicts, as is the case of the Anglophone crisis, it is wise to speedily resolve them so that the conflicts are not protracted. As the Anglophone crisis persists, the militia groups are gaining more experience at warfare and their diaspora sponsors are mobilising more funds to upgrade the weapons of the separatist fighters. Government’s anticipated military solution to the conflict is, therefore, implausible. We believe there is still enough time to avert a full-blown civil war in the Cameroons if the government can embark on a peaceful path towards resolving the crisis. This can rapidly be achieved through rational, sensible, reasonable, and inclusive political dialogue with the secessionist leaders, mediated by agreed-upon third parties.

This exploratory research has contributed to diagnosing the ramifications of the Anglophone crisis, determined the status of the crisis as acute complex emergencies, and proposed a new complex emergency paradigm (CDE). Nevertheless, there is an urgent need for in-depth scientific research to narrow down the time interval before the next Mt. Cameroon eruption. Further investigations on the potential dynamics and complexity that may arise when complex emergencies and disasters occur simultaneously and the implications for societal vulnerabilities would be beneficial to the humanitarian community. These areas of research would facilitate the building of resilience capacity and elicit a more timely and appropriate response to CDE-type scenarios.

## Data Availability

Not applicable.

## Abbreviations

AHA: Agence Humanitaire Africaine
CDE: Complex Disaster Emergency
CE-DAT: Complex Emergency Database
CFA: Communauté financière d'Afrique (French) or “Financial Community of Africa”
COVID-19: Coronavirus disease 2019
FAO: Food and Agriculture Organization
GRID: Global Report on Internal Displacement
GRINP: Gestion des Risques Naturels et Protection Civile (French) or “Natural Risk Management and Civil Protection”
HRW: Human Rights Watch
ICG: International Crisis Group
IMC: International Medical Corps
IMF: International Monetary Fund
IOM: International Organisation for Migration
PIC: Plan International Cameroon
UN Women: United Nations Entity for Gender Equality and the Empowerment of Women
UNOCHA: United Nations Office for the Coordination of Humanitarian Affairs
UNAIDS: United States Agency for International Development
UNDSS: United Nations Department of Safety and Security
UNDRR: United Nations Office for Disaster Risk Reduction
UNFPA: United Nations Population Fund
UNHCR: United Nations High Commission for Refugees
UNICEF: United Nations International Children’s Emergency Fund
USAID: United States Agency for International Development
WFP: World Food Programme
WHO: World Health Organization

## Footnotes

1 Following Law No 61-24 of September 21, 1961, the French and the British parts of Cameroon re-united with effect from October 1, 1961.

2 The Anglophones have always complained that ever since the union in 1961, the Francophones dominate the state executive, the legislature, and the judiciary including key institutions and government ministries.

3 The CVL is an alignment of 1600-km Y-shaped chain of volcanoes comprising an oceanic sector (Pagalu, Sao Tome, Principe, Bioko) and a continental sector (Mt. Etinde, Mt. Cameroon, Manengouba, Bambouto, Oku, Mandara, etc.) trending averagely N30°E through the country.

4 A recent mudslide from Mt. Cameroon (March 2020) kept the inhabitants of the neighbouring Buea town extremely worried, given the economic and psychosocial damage that emanated from it. This is just an indication if the possible disaster that can occur in case Mt. Cameroon erupts again.

5 Crises that last for several years result in a significant reduction in most human development indicators.

6 Crises that go through a series of phases, where peace and war alternate over an extended period.

7 A single country engulfed in different conflicts or have different conflict levels in its different regions.

8 All abbreviations have been written in full at the end of the article.

9 CE-DAT is a database established by more than 40 aid agencies containing data from more than 3186 different surveys that serves as a unique source of field data for monitoring the health status of conflict-affected populations. With over 20,000 health indicators, CE-DAT aims to share operational health data collected for planning, monitoring, and evaluation of humanitarian aid.

10 Spatial analysis is based on knowledge and characterisation of products of previous eruptions. It determines the position of vents, the existence of structural controls on vent distribution, and their spatial interrelations.

11 Temporal analysis refers to the eruption recurrence/frequency of a volcanic system, which can be determined via geochronology, stratigraphic, and historical data.

12 The Southern Cameroons Liberation Council is under the auspices of the self-declared Ambazonia Interim Government.

13 The Interim Government of Ambazonia is the leadership of the independence movement for the restoration of Ambazonia that is operating in exile. It was formed from the Southern Cameroons Ambazonia Consortium United Front (SACUF).

14 Institutions like the World Wide Web Foundation, Internet Sans Frontières, and Access Now were critical of government’s action to restrict Internet access in the Anglophone region.

15 The gruesome killing comprising 13 children and one pregnant woman led to international condemnation and several calls for the Government to respect and uphold human rights amidst the escalating conflict.

16 The Minister of Territorial Administration accused Amnesty International, Human Rights Watch, OCHA, and the International Crisis Group of colluding with anti-government forces and trying to destabilise state institutions.

17 The process required all humanitarian organisations to obtain permission to do a needs assessment before they can deliver aid to beneficiaries. The government also established Humanitarian Coordinating Centres in the capitals of the North West and South West regions to monitor incoming relief (distribution and traceability).

18 The teachers’ unions comprise of Cameroon Association of Teachers’ Trade Union, Teachers’ Association of Cameroon, Presbyterian Education Authority Teachers’ Trade Union, Baptist Teachers’ Trade union of Cameroon, Catholic Education Workers Trade Union of Cameroon, National Union of Teachers of Higher Education, University of Buea Chapter, and National Union of Teachers of Higher Education, University of Bamenda.

19 The Ordinary (O) Level and Advanced (A) Level are public examinations in Cameroon’s Anglophone region at the end of secondary and high school respectively set and marked by the Cameroon General Certificate of Education (GCE) Board.

20 Restriction on movement in the region has been caused by several random mobile road checkpoints mounted by both the security forces and non-state fighters.

21 75% of Cameroon’s arabica coffee comes from the North West Region.

22 The Upper Nun Valley Development Authority is in charge of developing the agricultural sector in the Upper Nun Valley of the NWR particularly with rice production, processing, and marketing, including providing support to the farmers.

23 This state-run agro-industrial company corporation is Cameroon’s second largest employer that has about 20,000 workers.

24 When interviewed on the famous radio programme “Cameroon Calling” on August 5, 2018, the general manager of the Cameroon Development Corporation said none of the 11 estates of the company was fully operational, adding that 4500, 5000, and 7000 jobs in the palm, banana, and rubber estates respectively were lost.

25 The CDD is a state-run agro-industrial company in Cameroon that has about 20,000 workers.

26 The eight chiefs kidnapped from Fako Division were the chiefs of Woteva, Wokwei, Wokeka, Wokaka, Ewili, Liwuh la Malale, and Lysoka.

27 Actors in the AC include the Government, military, security forces, civil society leaders, civilians, several armed groups and their sponsors in the diaspora, secessionists/federalist/unionist opinion groups, and political activist etcetera.
